# Exploring Bidirectional Associations Between Voice Acoustics and Objective Motor Metrics in Parkinson’s Disease

**DOI:** 10.3390/brainsci16010048

**Published:** 2025-12-29

**Authors:** Anna Carolyna Gianlorenço, Paulo Eduardo Portes Teixeira, Valton Costa, Walter Fabris-Moraes, Paola Gonzalez-Mego, Ciro Ramos-Estebanez, Arianna Di Stadio, Deniz Doruk Camsari, Mirret M. El-Hagrassy, Felipe Fregni, Tim Wagner, Laura Dipietro

**Affiliations:** 1 Neuromodulation Center and Center for Clinical Research Learning, Spaulding Rehabilitation Hospital, Mass General Brigham, Harvard Medical School, 1575 Cambridge Street, Cambridge, MA 02138, USA; gianlorenco@ufscar.br (A.C.G.); valtoncosta@estudante.ufscar.br (V.C.); walterafmoraes@gmail.com (W.F.-M.); paogonzalzm@gmail.com (P.G.-M.); fregni.felipe@mgh.harvard.edu (F.F.); 2 Laboratory of Neuroscience and Neurological Rehabilitation, Physical Therapy Department, Federal University of Sao Carlos, Rodovia Washington Luis, km 235, São Carlos 13506-900, SP, Brazil; 3 Highland Instruments, Inc., Cambridge, MA 02238, USA; paulo@highlandinstruments.com (P.E.P.T.); twagner@highlandinstruments.com (T.W.); 4 Faculty of Medicine, University of Sao Paulo, Avenida Doutor Arnaldo, 455, Sao Paulo 05508-090, SP, Brazil; 5 Department of Neurology and Rehabilitation, 912 S Wood St., Room 174N, MC 796, Chicago, IL 60612, USA; cramoses@uic.edu; 6 Department of Life and Health Science, Link University, 00100 Rome, Italy; ariannadistadio@hotmail.com; 7 Mayo Clinic, 200 1st St. SW, Rochester, MN 55901, USA; drdenizdoruk@gmail.com; 8 Mindpath College Health, 948 Embarcadero del Norte #102, Isla Vista, CA 93117, USA; 9 Neurology Department, UMass Memorial, UMass Chan Medical School, 67 Belmont St., Worcester, MA 01605, USA; mirret.elhagrassy@gmail.com; 10 Harvard/MIT Division of Health Sciences and Technology, Cambridge, MA 02139, USA

**Keywords:** Parkinson’s disease, voice analysis, voice biomarkers, motor performance, speech–motor interaction, wearable sensors

## Abstract

**Background/Objectives:** Speech and motor control share overlapping neural mechanisms, yet their quantitative relationships in Parkinson’s disease (PD) remain underexplored. This study investigated bidirectional associations between acoustic voice features and objective motor metrics to better understand how vocal and motor systems relate in PD. **Methods:** Cross-sectional baseline data from participants in a randomized neuromodulation trial were analyzed (n = 13). Motor performance was captured using an Integrated Motion Analysis Suite (IMAS), which enabled quantitative, objective characterization of motor performance during balance, gait, and upper- and lower-limb tasks. Acoustic analyses included harmonic-to-noise ratio (HNR), smoothed cepstral peak prominence (CPPS), jitter, shimmer, median fundamental frequency (F0), F0 standard deviation (SD F0), and voice intensity. Univariate linear regressions were conducted in both directions (voice ↔ motor), as well as partial correlations controlling for PD motor symptom severity. **Results:** When modeling voice outcomes, faster motor performance and shorter movement durations were associated with acoustically clearer voice features (e.g., higher elbow flexion-extension peak speed with higher voice HNR, β = 8.5, R^2^ = 0.56, *p* = 0.01). Similarly, when modeling motor outcomes, clearer voice measures were linked with faster movement speed and shorter movement durations (e.g., higher voice HNR with higher peak movement speed in elbow flexion/extension, β = 0.07, R^2^ = 0.56, *p* = 0.01). **Conclusions:** Voice and motor measures in PD showed significant bidirectional associations, suggesting shared sensorimotor control. These exploratory findings, while limited by sample size, support the feasibility of integrated multimodal assessment for future longitudinal studies.

## 1. Introduction

Parkinson’s disease (PD) is a progressive neurodegenerative disorder characterized by classic motor symptoms such as bradykinesia, rigidity, resting tremor, asymmetric onset, and postural instability (typically not in initial stages) [1]. At the movement level, these symptoms manifest as reduced movement amplitude and speed, impaired timing, increased movement variability, and reduced smoothness of voluntary movements, affecting upper-limb motor performance, gait, and postural control [2,3,4]. Beyond these hallmark motor features, PD also affects non-motor and additional motor domains, including fine motor coordination and speech [5,6]. Voice and speech impairments are among the most common and disabling symptoms of PD, with communication difficulties reported in up to 80% of patients [7,8,9,10,11]. These changes often appear early in the disease course and may be detectable even when global motor deficits are still mild [12].

Speech in PD is typically characterized by reduced intensity (loudness), monotone prosody, imprecise articulation, and phonatory instability, symptoms that define hypokinetic dysarthria [13]. Among these features, hypophonia, abnormally reduced vocal intensity during speech and sustained phonation, is a prominent and clinically salient manifestation, reflecting impaired amplitude scaling of the vocal motor output [11,14]. These speech abnormalities mirror core motor deficits observed in limb movement, such as reduced amplitude scaling (hypometria) and impaired motor coordination [8,15] and reflect deficits in the fine neuromotor control of respiratory, laryngeal, and articulatory subsystems. In PD, hypophonia typically manifests as a soft, weak, or poorly projected voice, with reduced vocal intensity and limited dynamic range and is commonly associated with bradykinetic laryngeal motor control and reduced respiratory–phonatory drive, rather than primary structural abnormalities of the vocal folds [11,16]. Importantly, both voice and limb movement are regulated by overlapping basal ganglia–cortical and cerebellar networks that coordinate timing and amplitude of motor output [17,18]. This shared neural architecture suggests that impairments in speech and motor control may emerge from common pathophysiological processes.

Auditory-perceptual evaluation is a core component of comprehensive voice assessment and is widely regarded as the clinical gold standard for characterizing voice quality in routine practice [19]. In clinical settings, clinicians frequently identify voice abnormalities through trained listening, often using standardized perceptual rating frameworks to guide interpretation [19]. However, despite its clinical utility, auditory-perceptual assessment remains inherently subjective and can be influenced by factors such as listener training and experience, rater expectations, and inter- and intra-rater variability, which may limit objectivity and reproducibility across evaluators and sites. Accordingly, acoustic analysis is commonly included as part of a full voice evaluation to provide complementary, quantifiable measures of vocal function and to support more standardized characterization of voice features.

Advances in acoustic signal analysis have made it possible to quantify voice alterations objectively and reproducibly. Acoustic metrics such as smoothed cepstral peak prominence (CPPS), jitter, and shimmer provide sensitive measures of phonatory clarity and stability, all of which are may be affected in PD and have been shown to relate to disease severity and treatment response [20,21]. At the same time, the use of instrumented motor assessment systems and wearable sensors enables the quantitative analysis of motor behavior, capturing fine-grained movement features such as movement speed, smoothness, variability, and postural sway, which provide objective indices of cardinal PD motor deficits including bradykinesia, impaired coordination, and postural instability [22,23,24]. These quantitative measures often outperform traditional clinical scales by detecting subtle deficits that may not be evident in standard examinations.

Despite these methodological advances, the relationship between voice and motor function in PD remains under characterized. Some studies have reported significant associations between vocal parameters and clinical motor severity, such as Unified Parkinson’s Disease Rating Scale motor scores (UPDRS-III) [25], as well as associations between voice measures (e.g., intensity, fundamental frequency (F0) variability, cepstral metrics) and specific motor domains such as gait and balance [26,27], while others have found weaker or no associations [28,29]. Studies conducted largely in the “On”-medication state report associations between motor domains and prosodic or phonatory measures, including reduced F0 variability and altered mean F0 in relation to axial impairment and gait-related features, as well as relationships between articulatory measures and rigidity, bradykinesia, and axial subscores [30,31,32]. Methodological differences, limited sample sizes, and the use of subjective rating scales rather than quantitative data likely contribute to this inconsistency. Recent work by Gianlorenço et al. (2024) [33] suggested that CPPS was associated with postural stability scores, also supporting the notion of a potential cross-domain link between voice and motor control. However, most studies have not examined whether these relationships are bidirectional, that is, whether motor impairments associate with voice features to the same extent that voice features reflect motor dysfunction. Examining this potential symmetry is important for clarifying whether voice and motor control share a bidirectional coupling in which the two domains dynamically influence each other through overlapping neural and sensorimotor circuits, or whether they simply co-vary as parallel but independent manifestations of overall disease severity. The term “bidirectional” is used here to denote statistical symmetry between modeling approaches rather than to imply causal direction.

To address this gap, we conducted an exploratory, proof-of-concept study modeling voice and motor measures in both directions. Depending on the strength and direction of the observed relationships, voice features might serve as non-invasive correlates of motor control, while motor measures could provide insight into speech-motor coupling and sources of vocal variability. Voice-related measures might also serve as biomarkers of PD progression and severity, as previously observed in Multiple Sclerosis (MS) [34].

To examine these relationships rigorously, we used the Integrated Motion Analysis Suite (IMAS), a multi-sensor platform that captures detailed kinematic data from balance, gait, and upper- and lower-limb tasks [22]. Unlike traditional clinical scales that rely on observer ratings, IMAS provides continuous, objective, and reproducible measurements of movement speed, variability, and coordination. This level of granularity enables the detection of subtle motor alterations that may correspond to fine vocal instabilities, offering a unique opportunity to study cross-domain relationships between speech and motor performance. We hypothesize that IMAS-based metrics, when compared to the UPDRS-III, will uncover subtle and biologically meaningful relationships between motor and vocal control that may not be detectable with clinical scales alone, thereby enabling a novel, bidirectional characterization of speech-motor coupling in PD.

This study examined the cross-sectional relationships between acoustic voice features (e.g., clarity, stability, and F0) and objective motor performance metrics derived from the IMAS (e.g., movement speed, variability, smoothness, and postural sway) in individuals with PD. Rather than assuming causality or directionality, the study aimed to describe how vocal and motor control co-vary within an integrated, exploratory framework. Notably, although PD shares clinical features with other degenerative neurological disorders and the measures used in this investigation are not pathognomonic, this study focuses on a clinically well-defined PD cohort to explore associations between voice and motor features rather than to establish diagnostic specificity.

## 2. Materials and Methods

### 2.1. Study Design

**Figure 1:**
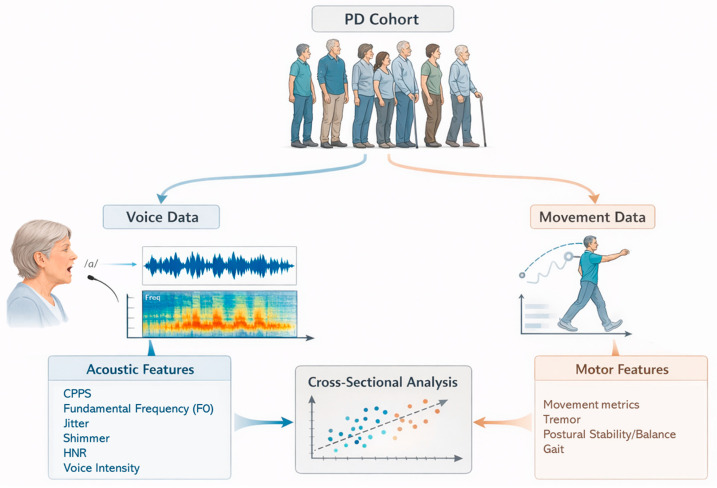
Simplified study design.

The dataset analyzed in this study was derived from baseline assessments conducted as part of a randomized controlled trial investigating the effects of non-invasive brain stimulation for the treatment of PD (ClinicalTrials.gov Identifier: NCT01615718), carried out at the Neuromodulation Center, Spaulding Rehabilitation Hospital, Charlestown, MA, USA. Not all the patients examined herein entered the main trial; this dataset consists of those who underwent baseline voice assessments. The study protocol was reviewed and approved by the Mass General Brigham Institutional Review Board, and written informed consent was obtained from all participants prior to enrollment. Figure 1 illustrates the study design.

### 2.2. Participants

Inclusion criteria required participants to have a diagnosis of idiopathic PD, be aged 40 years or older, and to have maintained stable medication regimens for at least 30 days prior to enrollment. Exclusion criteria included any contraindications to non-invasive brain stimulation, clinical features suggestive of atypical parkinsonian syndromes (PD-plus), unstable medical conditions, or a history of deep brain stimulation or ablative surgery. Thirteen participants from the main study completed the baseline voice assessments and were included in the present analysis. All participant assessments were initiated with the participants in their medication “On” states as detailed in [33].

### 2.3. The Unified Parkinson’s Disease Rating Scale Part III (UPDRS-III)

The UPDRS-III score [35] was used for assessment of motor symptoms, evaluating 14 motor domains through clinician-administered tasks, generating sub-scores for speech, facial expression, rest and postural tremor, rigidity, finger tapping, hand and rapid alternating movements, leg agility, arising from a chair, posture, gait, postural stability, and body bradykinesia.

### 2.4. Vocal Recording and Measures

Participants were recorded while sustaining the vowel/a/sound for a minimum of 9 s in a quiet testing environment and instructed to use their typical (habitual) loudness and pitch. Voice recordings were collected using a standard Windows Sound Recorder application with an external USB microphone, without an external preamplifier. The microphone was positioned at a close-talking distance in front of the participant’s mouth, at mouth level. Recordings were saved in uncompressed WAV format to preserve signal fidelity. To reduce potential artifacts from phonation onset and offset, only the central 5 s of each production were retained for analysis.

Acoustic features were extracted using Praat software (version 6.4.12, Institute of Phonetic Sciences, Amsterdam, The Netherlands), following validated procedures described in [36]. Recordings were pre-emphasized at 50 Hz and processed with Hann-windowed high-pass/low-pass filtering, with the analysis bandwidth set to 10–5000 Hz. These acoustic measures were analyzed to characterize vocal quality, phonatory stability, F0 characteristics, and vocal intensity and included CPPS, harmonic-to-noise ratio (HNR), jitter, shimmer, median F0, F0 standard deviation (SD F0), and overall voice intensity.

**Figure 2:**
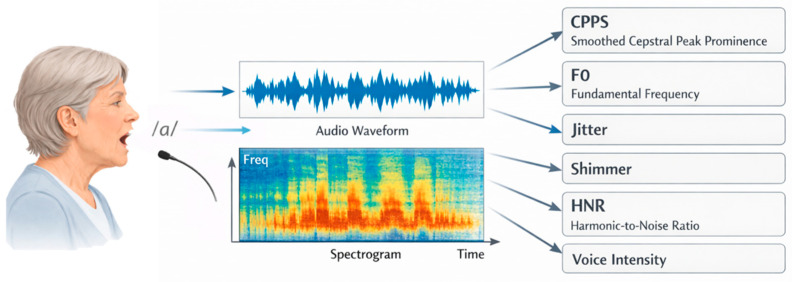
Vocal recording and measures.

CPPS was computed as the difference in amplitude between the fundamental frequency (F0) peak and a regression baseline in the cepstral domain, obtained via inverse Fourier transformation. CPPS reflected the degree of harmonic organization in the voice signal and is widely regarded as a robust indicator of dysphonia severity [37]. Jitter quantified short-term, cycle-to-cycle variability in F0, serving as a measure of temporal instability; higher jitter values indicated less regular vocal fold vibration [38,39]. Shimmer, expressed as a percentage, captured short-term amplitude perturbations and provided insight into amplitude stability, with larger values suggesting greater vocal irregularity [40]. The HNR, expressed in decibels (dB), reflected the relative energy of harmonic (periodic) versus noise (aperiodic) components of the voice signal, with higher values indicating clearer, more periodic phonation [41]. Figure 2 illustrates the vocal recording and measures.

Additional measures were included to provide a more comprehensive characterization of voice quality. Median F0 represented the central tendency of F0 distribution during sustained phonation [42]. SD F0 captured F0 variability, with higher values reflecting greater variation in F0 [43]. Voice intensity, expressed in decibels (dB SPL), measured the average intensity of the voice signal [44].

### 2.5. Integrated Motion Analysis Suite (IMAS)

The IMAS system for PD assessment is fully described in [22,45]. Briefly, IMAS is a portable, multi-sensor platform integrating a depth-camera, inertial, and force sensors. The system included a Kinect-based 3D camera for tracking 20 body joints, wearable inertial measurement units (IMUs; three-axis accelerometer and gyroscope) for capturing limb and trunk motion, and a portable force plate for balance assessment. Sensor configuration varied by task (see below) to optimize information capture while minimizing setup time. Recordings from all sensors were synchronized via custom software to ensure temporal alignment.

The data acquisition protocol comprised seven standardized tasks as described previously [22]. Upper-limb bradykinesia and movement control were evaluated through continuous and discrete elbow flexion–extension (Task 1A/B, 10 repetitions each) and hand opening–closing at both shoulder and waist levels (Task 2A/B, 10 repetitions each). These tasks yielded measures of movement duration, peak and mean speed, inter-movement intervals, and movement smoothness [22]. A multi-joint sequence combining elbow and hand movements (Task 3, 10 repetitions) was used to quantify complex motor coordination, with movement duration as the primary metric. Hand-to-nose pointing (Task 4, 10 repetitions) provided additional indices of speed, smoothness, and temporal consistency of goal-directed movements [22].

Tremor-related features were assessed under two conditions of sustained postures: with the arm relaxed on a table (Task 5A) and held in front of the face (Task 5B). Tremor power was quantified within specific frequency bands (3–6 Hz and 5–8 Hz), using both FFT- and multitaper-based spectral methods, expressed as relative power ratios [22].

Postural stability was examined with a modified Romberg test performed on a Nintendo Wii Balance Board (Task 6), once with eyes open and once with eyes closed. Metrics included standard deviations of center-of-pressure (CoP) displacement, path length (total distance traveled by the CoP trajectory), ellipse area (area of the fitted CoP ellipse), the lengths of its major and minor axis, and accelerometer-derived jerk measures characterizing sway dynamics. Finally, gait was evaluated with a 10 m walk repeated four times (Task 7), from which mean walk duration, stride count, stride time and variability, as well as accelerometer-derived jerk measures (mean, peak, and normalized), were obtained. For all tasks involving repeated movements (Tasks 1–4 and 7), means and standard deviations (SD) were computed [22].

### 2.6. Statistical Analysis

Descriptive statistics were used to summarize demographic and clinical characteristics. For continuous variables, means and SDs were reported when data followed a normal distribution, while medians and interquartile ranges (IQR) were used for non-normally distributed data. Categorical variables were presented as frequencies and percentages. Normality of numerical variables was assessed using histograms and the Shapiro–Wilk test.

Univariate linear regression analyses were conducted to examine the relationships between motor and voice metrics. In the first set of models, voice-related metrics (including CPPS, shimmer, jitter, HNR, median F0, SD F0, and overall voice intensity) were entered as dependent variables, with the IMAS-based metrics serving as independent variables. To assess the symmetry and robustness of these associations, the analyses were also performed in the opposite direction, treating the motor metrics as dependent variables and the voice metrics as independent variables.

Given the small sample size, only univariate models were used to avoid overfitting and to maintain interpretability of the findings. Statistical significance was determined using a two-tailed alpha level of 0.05. Partial correlation analyses [46,47] were also conducted to examine the associations between the biomechanical and voice variables after accounting for PD motor symptom severity. Specifically, the UPDRS-III score was entered as a control variable to remove variance attributable to disease severity.

All analyses were performed using Stata/SE version 17.0 (StataCorp LLC, College Station, TX, USA). Given the exploratory nature of the research question and the limited sample size, adjustments for multiple comparisons were not applied. The primary objective of these analyses was to identify patterns of association and generate hypotheses for future confirmatory studies. Results are presented in tabular form, with models rank-ordered by explained variance (R^2^) to highlight the strongest associations. Only findings that were statistically significant in both the univariate regression and the partial correlation analyses are reported.

## 3. Results

### 3.1. Participants Characteristics

The sample included 13 participants (10 males and 3 females) with a mean age of 62.9 ± 10.1 years. The median Hoehn and Yahr stage was 2.5 (IQR 2–2.5), indicating mild to moderate disease severity. Details on the inclusion criteria can be found in [33]. Based on motor phenotype classification, 2 participants were categorized as tremor-dominant, 3 as akinetic-rigid, and 8 as mixed type. The median of the total score on the UPDRS-III was 18 (IQR 14–27) points. Missing data were considered to be missing completely at random (MCAR). Statistical tests of MCAR were not performed due to limited sample size; however, inspection of missing-data patterns did not suggest systematic or variable-specific missingness [48]. Given the exploratory nature of the study and the small sample size, imputation methods were not applied for this particular analysis, as they could introduce bias and unstable estimates [49]. All analyses were therefore conducted using available-case data for each outcome. Descriptive statistics of UPDRS-III derived variables are presented in Table 1, while Table 2 shows all the voice-related variable descriptives. The names and corresponding definitions of all variables that showed significant associations in the results are listed in Table 3.

### 3.2. Associations Between Voice and Motor Related Variables

#### 3.2.1. Associations with Voice Outcomes

When voice variables were analyzed as dependent measures, several IMAS motor metrics showed significant associations (Table 4). Faster and more controlled movements were generally related to improved acoustic measures. For instance, hand-to-nose movement duration was negatively associated with overall voice intensity (β = −26.75, R^2^ = 0.40, *p* = 0.03), indicating that participants with longer hand-to-nose movement duration produced softer, less projected voices. Similarly, faster elbow flexion–extension movements (peak speed) were associated (β = 8.5, R^2^ = 0.56, *p* = 0.01) with clearer, less hoarse voice (voice HNR). However, postural control metrics showed a complex pattern: higher balance jerk (indicating greater instability) was associated with higher HNR and intensity, contrary to the upper-limb findings. Importantly, partial correlation analyses controlling for PD motor symptom severity (UPDRS-III total score) revealed that the direction of these associations remained consistent with the direction of the regression coefficient (β), and that the observed relationships were not driven by differences in motor symptom severity, as measured by the UPDRS-III score. Overall, individuals who have faster motor performance and shorter movement durations tended to exhibit acoustic patterns indicating improved vocal quality/clearer voice or more projected phonation, although these patterns were not consistent across all measures.

#### 3.2.2. Associations with Motor Outcomes

When motor variables were modeled as dependent outcomes, several acoustic features showed significant associations (Table 5). For example, higher HNR was associated with both higher speed in both continuous (mean speed: β = 0.040, R^2^ = 0.52, *p* = 0.018 and discrete (mean speed: β = 0.02, R^2^ = 0.47, *p* = 0.029) elbow flexion–extension tasks. Similarly, higher CPPS was associated with lower mean gait step duration—shorter walking cycle duration or faster gait (β = −0.63, R^2^ = 0.49, *p* = 0.011). Conversely, greater F0 variability (SD F0) was strongly linked to increased postural sway (Balance Jerk; R^2^ = 0.78). Consistent with these findings, partial correlations controlling for motor symptom severity (UPDRS-III) confirmed that these associations remained independent of motor symptoms severity and retained the same direction as the regression results. Overall, participants with improved vocal quality tended to show faster movements and shorter movement durations, whereas poorer vocal quality was associated with slower movements and longer movement durations. Greater F0 variability was associated with more irregular postural adjustments. Vocal intensity showed mixed associations across motor tasks, indicating that not all acoustic features followed the same pattern across outcomes.

Generally, the analyses demonstrated a consistent pattern of association between voice and motor domains. In both modeling directions, clearer acoustic voice features, such as higher HNR and higher CPPS, were linked with faster upper-limb motor performance and faster gait. Conversely, a more distorted or hoarse voice, reflected by lower spectral clarity, and greater F0 variability, was associated with longer movement durations, slower movements, and, particularly in the case of greater F0 variability, more irregular postural adjustments, although other acoustic features did not always follow this same pattern across tasks. However, postural control metrics exhibited distinct patterns, where vocal intensity and clarity were occasionally positively associated with jerk measures. Despite variability in association strength across tasks, the effects demonstrated a generally consistent direction, indicating that voice and motor performance measures share a meaningful degree of variance across both upper-limb and postural control domains.

## 4. Discussion

This exploratory study identified several significant associations between quantitative voice features and objective motor performance metrics in individuals with PD. Some vocal features showed associations with motor performance, with clearer vocal characteristics relating to faster movements and shorter movement durations, and poorer vocal quality relating to slower, longer movements, although these patterns were not consistent across all measures. Notably, in addition to clarity-related measures, voice intensity also showed significant associations with motor timing and postural control, although following a pattern distinct from HNR and CPPS (voice clarity and quality, respectively). These relationships were observed across multiple motor tasks, from elbow flexion-extension and hand-to-nose coordination to balance testing, suggesting that the link between speech and movement extends across functional domains. Similar patterns emerged regardless of model direction, whether voice variables were treated as independent measures or as outcomes, indicating a reciprocal association between the two systems. Importantly, all associations remained significant after controlling for PD motor symptom severity, confirming that these relationships were independent of disease motor severity effects.

The observed associations between acoustic and motor measures likely reflect shared neural mechanisms underlying speech and movement control in PD. Both functions depend on the integrity of basal ganglia-cortical and cerebellar circuits involved in regulating timing, coordination, and amplitude of movement [50,51,52]. Within these networks, dopaminergic depletion in the basal ganglia disrupts internal cueing, amplitude scaling, and temporal precision of motor output, while altered cerebellar contributions affect error correction, rhythmicity, and sensorimotor integration—processes essential for both limb movement and speech production [19,53,54]. Altered function within these networks may therefore contribute to concurrent deficits in motor control and vocal quality, although the associations observed here do not simply reflect overall disease severity, given their persistence after controlling for UPDRS-III. This interpretation aligns with neuroimaging evidence showing overlapping activation of the supplementary motor area, premotor cortex, basal ganglia, and cerebellum during both limb movement and speech production [18,55,56]. Although the cross-sectional design prevents causal inference, the bidirectional pattern observed suggests that disturbances in one domain, such as movement coordination, may be mirrored by subtle changes in vocal control, consistent with the idea of a shared timing and rhythm disturbance in PD [57,58].

Voice production depends critically on respiratory control; a previous study conducted in individuals with progressive MS [34] showed that disability severity was negatively correlated with expiratory time, which was positively correlated with phonation time; phonation time was also negatively correlated with dysarthria scores. In PD, altered diaphragm movement has been reported to vary with disease severity (as indexed by the Hoen and Yahr stage) [59], which could also impact voice characteristics and help contextualize the associations observed here. In addition, PD is associated with characteristic voice changes such as hypophonia, altered vocal quality, and reduced pitch and intensity variability, reflecting impaired laryngeal motor control. Voice changes in PD may therefore arise from both direct mechanisms (basal ganglia disfunction affecting laryngeal motor control) and indirect mechanisms (postural and muscular impairments impacting respiratory/diaphragmatic function) [60].

The relationship between postural jerk and vocal measures warrants caution. Higher jerk under the eyes-closed condition was associated with both higher HNR and greater vocal intensity, and vocal intensity also related to hand-movement timing. These findings may reflect increased task effort or compensatory motor adjustments rather than specific phonatory mechanisms. For example, increased vocal intensity may index heightened overall effort during challenging balance conditions, while elevated jerk may arise from compensatory strategies, fatigue, or task-specific constraints such as sensory deprivation rather than improved postural control [61,62]. Prior studies [63,64] indicate that increased jerk in PD may reflect attempts to compensate for proprioceptive deficits or axial rigidity rather than instability alone, but the present analysis cannot distinguish among these possibilities. Larger studies are needed to clarify whether the observed association reflects compensatory motor behavior, rigidity-related mechanisms, or other factors.

The present findings highlight the close interdependence between speech and motor systems in PD. Viewing these associations bidirectionally underscores that both domains may reflect shared underlying motor control mechanisms rather than one serving strictly as a predictor of the other. This perspective aligns with emerging evidence that sensorimotor timing, coordination, and feedback integration are distributed across overlapping cortical-subcortical networks encompassing the basal ganglia, cerebellum, and premotor regions [18,50]. From a clinical standpoint, understanding this interrelation opens opportunities for multimodal assessment frameworks. For instance, acoustic measures could serve as accessible correlates of motor control for remote or continuous monitoring, while detailed motor kinematic data might help interpret or stratify speech-motor impairments [25,33,65,66]. These insights may also inform physical therapy and rehabilitation strategies, where real-time acoustic or kinematic feedback could potentially enhance motor learning and cueing-based interventions in PD, improving the coordination between movement and speech production [67,68]. Developing such integrative approaches could enhance quantitative monitoring strategies, enabling more precise evaluation of motor and speech-related changes during disease progression or treatment.

A key strength of this study lies in its use of objective, instrument-based quantification of both motor and voice domains. The IMAS system provided high-resolution kinematic and kinetic data that captured subtle features of motor control, such as movement speed, and variability, while acoustic analysis yielded reproducible measures of voice quality. This dual quantitative approach minimizes subjective bias inherent in clinical rating scales and allows for more precise characterization of motor and speech performance. The data were collected within a standardized clinical trial framework, ensuring consistent assessment conditions and well-characterized participant profiles. These features enhance the internal validity of the findings and support their potential applicability to rehabilitation contexts, where objective metrics can complement clinical observation, track therapy effects, and inform individualized intervention planning. Moreover, the reproducibility and scalability of these quantitative methods make them suitable for integration into longitudinal and multisite studies, extending their translational potential for clinical practice and research.

This study has limitations that should be considered when interpreting the findings. The cross-sectional design prevents conclusions about causality or temporal direction between voice and motor variables. Longitudinal data would be necessary to determine whether changes in one domain precede or mirror changes in the other. The small sample size limits statistical power and may have constrained the ability to detect weaker associations, particularly after accounting for interindividual variability. As a result, the findings should be interpreted as exploratory and may have limited generalizability. Only univariate models were applied, and potential confounding factors such as age, sex, and disease duration were not adjusted for, which may influence the observed relationships [69,70,71]. The voice and motor assessments were performed under separate task conditions, preventing direct temporal alignment between the two modalities and limiting the ability to assess their dynamic interaction. Sex-related effects on F0 were not examined due to the limited sample size and should be addressed in future studies with larger cohorts. Audiometric assessments were not performed, and therefore potential contributions of peripheral hearing status to acoustic measures could not be evaluated. While the audio acquisition hardware used in this study was not studio-grade, recordings were obtained using a consistent hardware and software configuration throughout data collection. Future iterations of this work are planned to incorporate higher-grade audio recording equipment, which may further improve signal fidelity and generalizability across recording environments. Finally, because multiple comparisons were conducted without formal correction in this exploratory study, the results may reflect inflated Type I error. Statistical findings are presented cautiously and should not be interpreted as confirmatory. Despite these limitations, the consistency of the results across analytic directions supports the robustness of the associations and provides a valuable foundation for more comprehensive, hypothesis-driven investigations.

## 5. Conclusions

This exploratory study identified significant associations between quantitative voice features and objective measures of motor performance in individuals with PD. Clearer vocal characteristics were generally associated with faster and more efficient movements, while poorer vocal quality related to slower performance; voice intensity showed distinct associations with motor timing and postural control. These associations were observed across multiple motor tasks and remained significant after controlling for overall motor severity. Future work should build on this exploratory study by following participants over time to see whether changes in voice and motor performance occur together or respond similarly to treatment. Longitudinal designs would help determine if changes in one domain can predict or reflect improvements in the other. Studies that record voice and motor signals at the same time would also make it possible to test how closely these systems fluctuate together during specific tasks. These approaches could be applied in rehabilitation and neuromodulation trials, where objective voice and movement data may help monitor progress and guide individual therapy. Combining these quantitative measures with imaging or physiological data could also improve understanding of how speech and movement share neural control in PD. Overall, this line of research supports developing integrated and objective assessment methods that describe motor function and treatment effects more clearly than single-domain measures.

## Figures and Tables

**Table 1:** Descriptive statistics for UPDRS-III derived variables.

UPDRS-III-Derived Variables (Domains)	Mean ± SD
Speech	1.2 ± 0.9
Facial expression	1.2 ± 1.0
Rigidity	3.2 ± 2.0
Finger tapping	1.8 ± 1.2
Hand movements	1.9 ± 1.5
Alternating movements	1.8 ± 1.5
Leg agility	1.8 ± 1.7
Posture	1.1 ± 0.8
Gait	0.8 ± 0.7
Postural stability	0.7 ± 0.6
Bradykinesia	1.5 ± 1.0
Arising from chair *	0 (0)
Kinetic Tremor *	2.0 (1–2)
Rest Tremor *	1.0 (0–3)

* Reported as Median and IQR (data not normally distributed).

**Table 2:** Descriptive statistics for voice-related variables.

Variable	Obs	Mean	SD	Min	Max
CPPS	12	11.75	2.86	5.06	15.75
Median F0	10	152.54	31.21	97.27	203.49
Jitter	10	0.67	0.37	0.27	1.53
Voice Shimmer	10	6.56	2.12	2.75	10.02
HNR	10	21.11	5.94	10.36	32.75
SD F0 *	10	4.6	3.48	2.04	19.96
Voice Intensity *	12	48.1	7.47	32.49	68.23

Abbreviations: Obs: Number of observations; Min: Minimum; Max: Maximum; CPPS: Smoothed cepstral peak prominence; HNR: Harmonic-to-noise ratio; SD: Standard deviation. * Reported as Median and IQR (data not normally distributed).

**Table 3:** Names and meanings of all variables that exhibited significant associations in the results.

**Voice Variables**	**Meaning/Interpretation**
Voice_HNR	Ratio of harmonic (periodic) to noise (aperiodic) energy in the voice. Higher values = clearer, less breathy/hoarse voice.
CPPS	Degree of harmonic organization in the voice signal. Higher values = improved voice quality (greater cepstral prominence).
Voice_Intensity	Overall intensity (loudness) of the voice (vocal projection). Higher values = louder voice.
Voice_F0_SD	F0 variability (standard deviation of F0). Lower values = less variation in vocal frequency (more stable F0); Higher values = greater variation in vocal frequency (less stable F0).
**IMAS (Motor) Variables**	**Task/Meaning**
Balance_Jerk_EC	Eyes closed balance test—peak jerk amplitude. Higher values = greater postural adjustment irregularity.
Balance_Jerk_EO	Eyes open balance test—peak jerk amplitude. Higher values = greater postural adjustment irregularity.
Elbow Flex/Ext _PeakSpeed	Continuous elbow flexion/extension movements—peak speed of movement (average across movements).
Elbow Flex/Ext _MeanSpeed	Continuous elbow flexion/extension movements—mean speed of movement (average across movements).
Elbow Flex/Ext_MeanSpeed_SD	Continuous elbow flexion/extension movements—standard deviation for mean speed of the Elbow Flex/Ext movement.
Gait_StepDur_Mean	Walking test—average step duration. Higher values = slower gait.
Gait_StrideCount_Mean	Walking test—stride count. More strides = shorter steps.
ElbowDisc_MeanSpeed	Discrete elbow flexion/extension movements—mean speed of movement (average across movements).
HandSqueeze_InterTime	Hand opening/closing task—time between squeezes. Smaller values = faster repetition rate (average across movements).
HandNose_MoveDur	Hand-to-nose—mean duration of movement (average across movements).

**Table 4:** Univariate linear regression analyses using voice-related variables as dependent variables.

Dependent Variable (Voice)	Independent Variable (Motor)	R^2^	β Coefficient	*p*-Value	Interpretation	Pcorr	*p*-Value (Pcorr)
Voice_F0_SD	Balance_Jerk_EO	0.78	2.72	0.001	Higher balance jerk (eyes open, greater postural adjustment irregularity) → greater variation in vocal frequency (less stable F0).	0.8806	0.0017
Voice_HNR	ElbowFlex/Ext_PeakSpeed	0.56	8.50	0.013	Faster elbow flexion–extension movements → clearer, more periodic phonation.	0.7601	0.0174
Voice_HNR	ElbowDisc_MeanSpeed	0.47	25.00	0.029	Faster discrete elbow flexion–extension speed → clearer, more periodic phonation.	0.6669	0.0498
Voice_HNR	Balance_Jerk_EC	0.41	1.89	0.045	Higher balance jerk (eyes closed, greater postural adjustment irregularity) → clearer, more periodic phonation.	0.6759	0.0457
Voice_Intensity	HandNose_MoveDur	0.40	−26.75	0.027	Longer hand-to-nose movement duration → lower vocal intensity.	−0.6389	0.0343
Voice_Intensity	HandSqueeze_InterTime	0.39	44.14	0.029	Longer time between hand squeezes → higher vocal intensity.	0.7071	0.0149
Voice_Intensity	Balance_Jerk_EC	0.36	3.16	0.038	Higher balance jerk (eyes closed, greater postural adjustment irregularity) → higher vocal intensity.	0.6103	0.0462

**Table 5:** Univariate linear regression analyses using IMAS related variables as dependent variables.

Dependent Variable (Motor)	Independent Variable (Voice)	R^2^	β	*p*-Value	Interpretation of Direction	Pcorr	*p*-Value (Pcorr)
Balance_Jerk_EO	Voice_F0_SD	0.78	0.29	0.001	Greater variation in vocal frequency (less stable F0) → higher balance jerk (eyes open, greater postural adjustment irregularity).	0.8806	0.0017
Gait_StrideCount_Mean	CPPS	0.58	−0.59	0.004	Higher CPPS values, indicating improved vocal quality → lower stride count (fewer, longer steps to cover the 10 m).	−0.7081	0.0148
ElbowFlex/Ext_PeakSpeed	Voice_HNR	0.56	0.070	0.013	Clearer, more periodic phonation → higher peak speed in continuous elbow flexion/extension movements.	0.7601	0.0174
ElbowFlex/Ext_MeanSpeed	Voice_HNR	0.52	0.040	0.018	Clearer, more periodic phonation → faster continuous elbow flexion/extension movements.	0.7186	0.0292
Gait_StepDur_Mean	CPPS	0.49	−0.63	0.011	Higher CPPS values, indicating improved vocal quality → shorter walking cycle duration (faster gait).	−0.6285	0.0383
ElbowDisc_MeanSpeed	Voice_HNR	0.47	0.020	0.029	Clearer, more periodic phonation → faster discrete elbow flexion/extension movements.	0.6669	0.0498
Balance_Jerk_EC	Voice_HNR	0.41	0.220	0.045	Clearer, more periodic phonation → higher balance jerk (eyes closed, greater postural adjustment irregularity).	0.6759	0.0457
HandNose_MoveDur	Voice_Intensity	0.40	−0.01	0.027	Higher vocal intensity → shorter hand-to-nose movement duration (faster movements).	−0.6389	0.0343
ElbowFlex/Ext_MeanSpeed_SD	CPPS	0.39	0.020	0.030	Higher CPPS values, indicating improved vocal quality → slightly greater variability in mean speed in continuous elbow flexion/extension movements.	0.6998	0.0165
HandSqueeze_InterTime	Voice_Intensity	0.39	0.010	0.029	Higher vocal intensity → longer time between squeezes (slower hand opening/closing repetition rate).	0.707	0.0149
Balance_Jerk_EC	Voice_Intensity	0.36	0.110	0.038	Higher vocal intensity → higher balance jerk (eyes closed, greater postural adjustment irregularity).	0.6103	0.0462

## Data Availability

The original contributions presented in this study are included in the article. Further inquiries can be directed to the corresponding authors.

## Abbreviations

The following abbreviations are used in this manuscript:

Acronym	Full Term
CPPS	Cepstral Peak Prominence Smoothed
dB	Decibels
F0	Fundamental Frequency
HNR	Harmonic-to-Noise Ratio
IQR	Interquartile Range
IMAS	Integrated Motion Analysis Suite
PD	Parkinson’s Disease
SD	Standard Deviation
UPDRS-III	Unified Parkinson’s Disease Rating Scale Part III
EC	Eyes Closed
EO	Eyes Open
